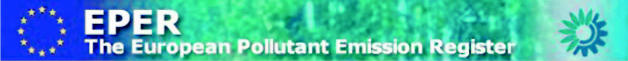# EHPnet: European Pollutant Emission Register

**Published:** 2004-08

**Authors:** Erin E. Dooley

Pollutant release and transfer inventories are a relatively new database-driven means of providing information on the who, what, and how much of industrial emissions. Though governments have for some time collected such data for their own use, it has only been in the last decade or so that a move has been under way to make this information publicly available. Agenda 21, the plan of action adopted at the 1992 United Nations Conference on Environment and Development, advocated the development of national registries in each of the participating countries as a means of educating the general public and others about pollution sources. Today, inventories of emissions from more than 9,000 large and medium facilities in 16 European countries are available for free online through the European Pollutant Emission Register (EPER), located at **http://www.eper.cec.eu.int/eper/**.

A joint project of the European Commission and the European Environment Agency, EPER allows users to compare data between such variables as industry type and locale so that interested parties can act to reduce disparities. Environment commissioner Margot Wallström commented at the 23 February 2004 launch of the register that people need to know about pollution in their environment because it directly affects their health and their quality of life. She added that by using the register, citizens can put pressure on government and industry—an essential aspect of the public’s involvement in protecting the environment.

The data included within EPER have been provided by facilities that exceed specified emission thresholds. The data cover 50 air and water pollutants that can harm human and environmental health, including arsenic, lead, mercury, nitrogen, phosphorus, and small particulate matter. Industrial sectors include pig and poultry farming, minerals, metals, pharmaceuticals, cement and glass, asbestos, and waste disposal. The current version of EPER includes data from the year 2001; a set of year 2004 data will be added in 2006.

Choosing the Facility Level search allows users to search for facilities by area—all of the European Union countries or any of 17 individual nations. Users can also choose from pull-down lists of pollutants and industrial activities, and search by facility name and/or address. Users can also choose to run an Industrial Activity or Pollutant search.

The Map Search tool of the website allows the user to create a customized color-coded map that can show such elements as the density of all EPER industries across a region, the density of certain types of industries in a certain area, and the industries in a single metropolitan area. The map can also be configured to show only facilities emitting a single substance across specified areas.

EPER has also provided a searchable glossary of terms related to industry and pollutants. Links to the national emissions registers that were used in helping to compile the EPER database, as well as to a number of European and international environmental organizations, are available as well, under the Links heading.

## Figures and Tables

**Figure:**